# Intertumoural Heterogeneity and Branch Evolution of Synchronous Multiple Primary Lung Adenocarcinomas by Next-Generation Sequencing Analysis

**DOI:** 10.3389/fonc.2021.760715

**Published:** 2021-11-03

**Authors:** Qinleng Zhang, Hui Jia, Zhendan Wang, Shaoyu Hao, Haiyan Huang, Airong Yang, Lu Han, Pingping Song

**Affiliations:** ^1^ Shandong First Medical University and Shandong Academy of Medical Sciences, Jinan, China; ^2^ Department of Thoracic Surgery, Shandong Cancer Hospital and Institute, Shandong First Medical University and Shandong Academy of Medical Sciences, Jinan, China; ^3^ Department of Respiratory Internal, Shandong Cancer Hospital and Institute, Shandong First Medical University and Shandong Academy of Medical Sciences, Jinan, China; ^4^ Department of Bioinformatics, Berry Oncology Corporation, Beijing, China

**Keywords:** somatic mutation, trunk-branch evolution, intertumoural heterogeneity, single-nucleotide variants, synchronous multiple primary lung adenocarcinomas

## Abstract

**Objectives:**

Multiple primary lung cancers (MPLCs) are an increasingly well-known clinical phenomenon, but there is a lack of high-level evidence for their optimal clinical diagnosis and therapeutic approaches. Thus, we analysed genetic variation to determine the intertumoural heterogeneity and branch evolution of synchronous multiple primary lung adenocarcinomas.

**Methods:**

We performed multiplex mutational sequencing on 93 synchronous multiple primary lung adenocarcinoma lesions from 42 patients who underwent surgical resection.

**Results:**

The high discordance rate of mutation was 92.9% (n=39) between tumours in individual patients. EGFR, TP53 and KRAS mutations were detected in 57 (61.3%), 19 (20.4%) and 11 (11.8%) of the 93 tumours, respectively. 16 cases of multiple primary lung adenocarcinomas simultaneously harboured EGFR mutations and TP53 mutations. Matching mutations between paired tumours were observed in 1 (2.4%) patient for P20. The genotypes were all EGFR L858R mutations, but the pathological type of P20T1 was lepidic predominant, and P20T2 was adenocarcinoma in situ. In the phylogenetic tree, genetic variations were divided into trunk, shared and branch subtypes. Branch mutations accounted for 91.09% of variations in sMPLA, while the ratio of trunk (4.95%) and shared (3.96%) variations was significantly lower.

**Conclusions:**

Remarkable intertumoural heterogeneity and frequent branch mutations were found in synchronous multiple primary lung adenocarcinomas.

## Introduction

Lung cancer remains the leading cause of cancer incidence and mortality in most countries (1). Due to advances in imaging diagnostic technology and the emphasis on early lung cancer screening, synchronous multiple primary lung cancers have been detected more frequently by multislice spiral computed tomography (CT) and positron emission tomography (PET) scanning, ranging from 0.2% to 20% (2). Martini and Melamed established the first multiple primary lung cancer (MPLC) diagnostic criteria in 1975 and proposed that tumours were ‘synchronous’ when they were detected or resected simultaneously (3), which was later revised by the American College of Chest Physicians (ACCP) (4, 5). Chen et al. demonstrated that adenocarcinoma-adenocarcinoma was the most common pathological type in multiple primary lung cancers (6).

Multiple primary lung cancers with different molecular characteristics are heterogeneous diseases (7). Heterogeneity in cell morphology, proliferation rate, metastatic ability, drug sensitivity, dependence on growth signals, and tumour initiation ability has been considered a significant feature of most malignant tumours (8). As a potential cause of drug resistance in targeted therapy, lung cancer heterogeneity may promote tumour evolution and adaptation and decrease sensitivity to individualized treatment (9).

Next-generation sequencing technology has facilitated advances in our understanding of genetic and epigenetic tumour heterogeneity (10). The trunk-branch model was used to explain tumour heterogeneity in the phylogenetic tree. Trunk gene mutations are usually early somatic genetic alteration events that drive tumour growth in each tumour region. As the disease progresses, branch gene mutations may appear in primary tumours and/or metastases, causing intertumoural heterogeneity or intratumoural heterogeneity (11).

In previous research, genomic analysis was used to determine the extent to which multifocal lung cancers share the same ancestor, providing a basic theory for the evolutionary principle of tumorigenesis. However, the use of multigene sequencing to analyse molecular cloning relationships between synchronous lesions in synchronous multiple primary lung adenocarcinoma (sMPLA) is relatively rare. We used next-generation sequencing technology to elucidate the intertumoural heterogeneity among synchronous multiple primary lung adenocarcinoma lesions and judge the clonal mutation frequency of the lesion.

## Materials and Methods

### Patients

Between June 2018 and March 2021, a total of 42 sMPLA patients underwent surgery at the Tumor Hospital affiliated to Shandong First Medical University in Jinan, China. These patients did not receive neoadjuvant radiotherapy and chemotherapy or were diagnosed with intrapulmonary and extrapulmonary metastases. Synchronous multiple primary lung adenocarcinoma was defined as the occurrence of a second lung adenocarcinoma within 6 months of the first lung adenocarcinoma (12). The TNM stage of lung cancer was determined according to the International Association for the Study of Lung Cancer (IASLC) staging system, version 8. This study was approved by the Ethics Committee of the Tumor Hospital Affiliated to Shandong First Medical University. Our research team obtained written informed consent from each participant before commencing any procedures related to the study. Pack-years is the number of packs per day multiplied by the number of smoking years.

### Histologic Evaluation

Two experienced lung cancer pathologists reviewed all pathological slides of each tumour and reclassified the adenocarcinoma according to the 2015 WHO classification on the basis of predominant histologic subtype. Each tumour was reviewed using comprehensive histologic subtyping, and the percentage of each histological component was semiquantitatively recorded in 5% increments (13). The predominant pattern that constituted the largest percentage of the histopathological examination was determined as the histological subtype. Each tumour was classified as adenocarcinoma *in situ* (AIS), minimally invasive adenocarcinoma (MIA), and invasive adenocarcinoma (IA), which were further divided into lepidic predominant (lepidic), acinar predominant (Acinar), papillary predominant (Papillary), micropapillary predominant (Micropapillary), and solid predominant (Solid) (13).

### Radiological Diagnosis

Two radiologists independently reviewed the TSCT scan to examine the appearance of lesions. The evaluated factors in the lung window were the maximum diameters of the tumour and consolidation. The consolidation component was defined as an area of increased opacification that completely obscured the underlying vascular markings. GGO was defined as an area of a slight, homogenous increase in density that did not obscure the underlying vascular markings. The radiologist assessed the maximum diameter and consolidation of the lesion through the lung window using axial plane CT scans. Our team selected two cut-off values of the CTR ratio in the lung window to predict the mutation characteristics of synchronous multiple primary lung adenocarcinomas (0.25 and 0.5) (14, 15).

### DNA Isolation, Sequencing, and Identification of Somatic Variants

Actionable gene mutation analysis of surgery tissue samples was determined by capture single molecule amplification and resequencing technology (capSMART) for a targeted NGS panel assay of 10 NSCLC-related driver genes (Berry Oncology, Beijing, China). Briefly, genomic DNA from postsurgery tumour tissue specimens and white blood cells was extracted according to the standard protocol provided in the DNeasy Blood & Tissue kit (Qiagen). The concentration of the purified DNA was measured by the Qubit^R^ dsDNA HS Assay Kit (Life Technologies, Grand Island, NY, United States). Fifty nanograms of DNA was fragmented to an average size of 300 bp in NEBNext dsDNA fragmentase buffer (New England Biolabs, MA, United States). DNA libraries were constructed as previously described (16), and hybridization capture of exonic regions from EGFR, ALK, ROS1, BRAF, RET, MET, ERBB2, KRAS, PIK3CA, TP53 and select introns from ALK, ROS1, RET commonly rearranged in cancer was applied. The target-enriched library was then paired end (PE) sequenced (2 × 150 bp) on the NovaSeq platform (Illumina) according to the manufacturer’s instructions with high, uniform median coverage (>1000×) and assessed for base substitutions, short insertions and deletions, copy number alterations, and gene fusions/rearrangements (16).

### Statistical Analyses

All statistical analyses were carried out with SPSS version 23.0 (SPSS, Inc., Chicago, IL). The descriptive statistics used included medians and ranges for continuous variables and percentages for categorical variables. Wilcoxon rank-sum test, Chi-square test and Fisher’s exact test were performed when rate or percentage was compared for significance. All P-values were two-sided, and P-values of less than 0.05 were considered statistically significant. R software was used to make tumour spectrum figures (https://www.r-project.org/).

## Results

### Patient Characteristics

**Figure 1:**
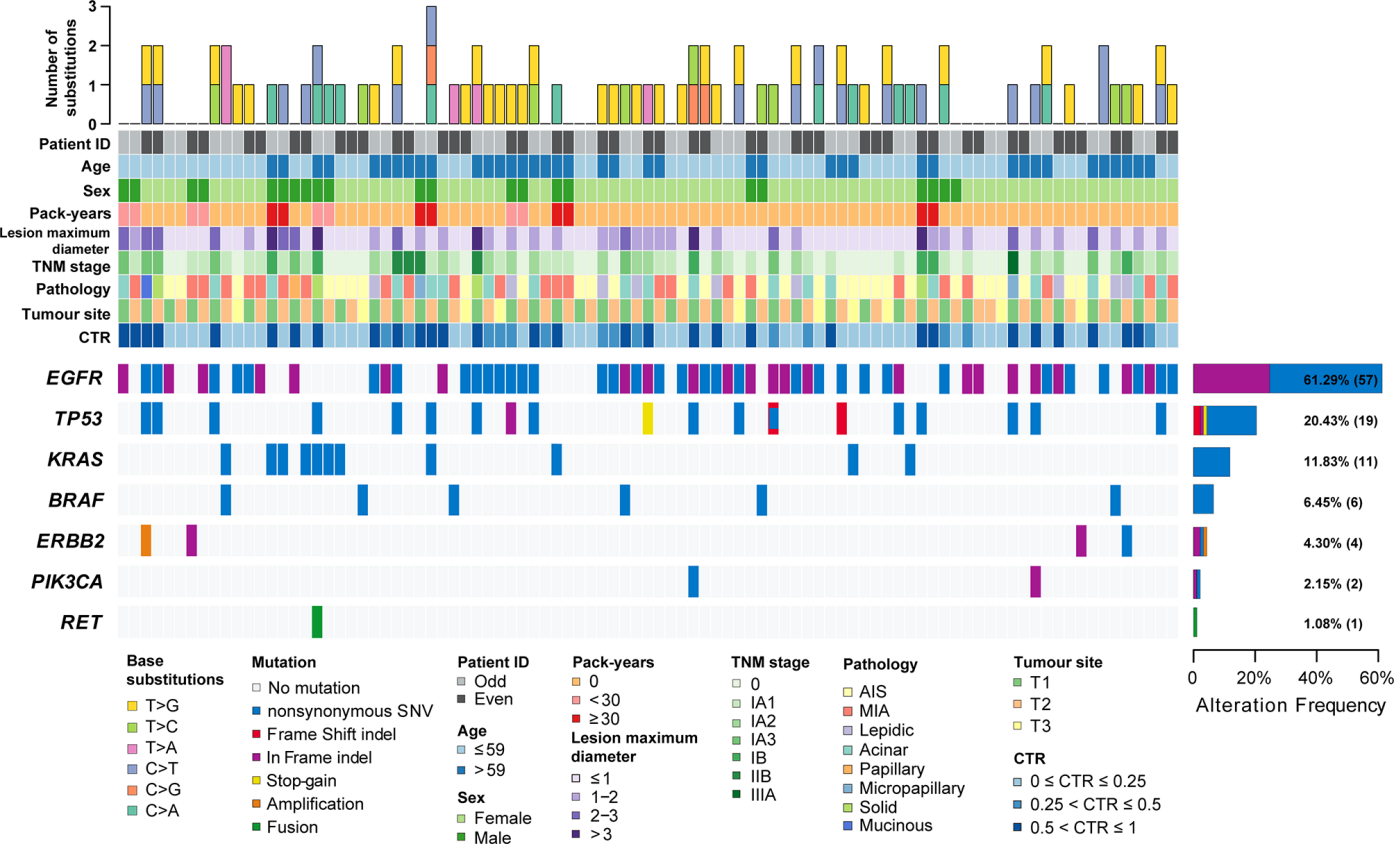
The clinicopathological and imaging features and mutation landscape of 93 synchronous multiple primary lung adenocarcinomas. The number and types of base substitutions in each tumour are shown in the upper panel. Clinical features are annotated in the middle panel. The heat map below clearly displays the number and type of somatic mutated genes in each tumour, including nonsynonymous SNVs (single nucleotide variants), frame shift indels, in-frame indels, stop-gain mutations, amplification and fusion.

The characteristics of 93 tumors from 42 patients with synchronous multiple primary lung adenocarcinomas (sMPLA) included in the study were summarized in **Table 1**. The patients were predominantly female (n=31 [73.8%]) and nonsmokers (n=34 [81.0%]), and their median age was 58 years (range 33–75 years). In 12 (28.6%) patients, multiple tumours were located in the same lobe, 26 (61.9%) patients had a tumour in the ipsilateral lobe, and 4 (9.5%) patients had a tumour in the contralateral lobe (**Table 1**). There were 33 cases with two tumours in the same patient and 9 cases with three tumours (**Figure 1**). In 37 patients, the size of the largest tumour was 3 cm or less, and tumours larger than 3 cm were detected in 5 cases (**Figure 1**).

**Table 1:** Clinical, pathological and imaging characteristics of 42 sMPLA cases.

Variables	Value
Patient characteristics (N = 42)	
Sex	
Female	31 (73.8%)
Male	11 (26.2%)
Mean age at first resection, y (range)	58 (33-75)
Pack-years, n (%)	
0	34 (81.0%)
0-30	4 (9.5%)
≥30	4 (9.5%)
Distribution of tumours, n (%)	
Ipsilateral (same lobe)	12 (28.6%)
Ipsilateral (other lobe)	26 (61.9%)
Contralateral	4 (9.5%)
Tumor characteristics (N = 93)	
Type of resection, n (%)	
Wedge resection	39 (41.9%)
Segmentectomy	10 (10.8%)
Lobectomy	44 (47.3%)
Location, n (%)	
LUL	22 (23.7%)
LLL	15 (16.1%)
RUL	26 (28.0%)
RML	11 (11.8%)
RLL	19 (20.4%)
CTR, n (%)	
0-0.25	49 (52.7%)
0.25-0.5	14 (15.1%)
0.5-1	30 (32.3%)
Pathological type, n (%)	
AIS	32 (34.4)
MIA	25 (26.9%)
Lepidic	9 (9.7%)
Acinar	19 (20.4%)
Papillary	1 (1.1%)
Micropapillary	2 (2.2%)
Solid	4 (4.3%)
Mucinous	1 (1.1%)
Node involvement, n (%)	
N0	90 (96.8%)
N1/2	3 (3.2%)
Pathological stage, n (%)	
0	32 (34.4%)
IA	50 (53.7%)
IB	6 (6.5%)
IIB	4 (4.3%)
IIIA	1 (1.1%)

sMPLA, synchronous multiple primary lung adenocarcinoma; LUL, left upper lobe; LLL, left lower lobe; RUL, right upper lobe; RML, right middle lobe; RLL, right lower lobe; AIS, adenocarcinoma in situ; MIA, minimally invasive adenocarcinoma; Lepidic, lepidic-predominant; Acinar, acinar-predominant; Papillary, papillary-predominant; Micropapillary, micropapillary-predominant; Solid, solid-predominant; CTR, consolidation to tumour ratio.

### Clinicopathological and Molecular Characteristics of Tumours

A total of 93 tumours were resected from 42 patients with sMPLA. The characteristics of the tumours are summarized in **Table 1** and **Figure 1**. Surgical procedures included 39 wedge resections, 10 segmentectomy resections, and 44 lobectomy resections. In 3 cases, N1 (n=2) and N2 (n=1) lymph nodes were positive. The pathological stage was 0 in 32 tumours (34.4%), IA1 in 27 tumours (29.0%), IA2 in 15 tumours (16.1%), IA3 in 8 tumours (8.6%), IB in 6 tumours (6.5%), IIB in 4 tumours (4.3%), and IIIA in 1 tumour (1.1%). According to the 2015 World Health Organization Classification of Lung Tumours, 34.4% of tumours were AIS (n=32), and 26.9% were MIA (n=25). The most common histologic subtype was acinar predominant (20.4%; n=19), followed by lepidic predominant (9.7%; n=9), papillary predominant (1.1%; n=1), micropapillary predominant (2.2%; n=2), solid predominant (4.3%; n=4), and mucinous predominant (1.1%; n=1) (**Table 1**).

EGFR kinase domain mutations were identified in 61.3% of the tumours (57 of 93; 95% CI, 0.512 to 0.714). Among these, 18 were deletions in exon 19, and 31 were L858R mutations (**Supplementary Table 1**). HER2 “YVMA” insertion mutations were seen in 2.2% (2 of 93; 95% CI, -0.009 to 0.052) of the samples. Similarly, 4.3% (4 of 93; 95% CI, 0.001 to 0.085) of the samples had a KRAS G12C mutation. A total of 1.1% (1 of 93; 95% CI, -0.011 to 0.032) of samples had a BRAFV600E mutation. A total of 1.1% (1 of 93; 95% CI, -0.011 to 0.032) of samples had an A1CF-RET fusion (**Supplementary Table 1**). TP53 mutations were observed in 20.4% of tumours (19 of 93; 95% CI, 0.121 to 0.288) (**Figure 1**). Additionally, 2.2% (2 of 93; 95% CI, -0.009 to 0.052) of samples were found to have a PIK3CA mutation. ALK fusions, ROS1 fusions and MET copy number variation were not found in these samples. A total of 17.2% (16 of 93; 95% CI, 0.094 to 0.250) of tumours harboured both EGFR mutations and TP53 mutations (**Supplementary Table 1**).

### Correlation of EGFR/KRAS/TP53 Mutations With Clinicopathological and CTR Characteristics

EGFR mutations were more frequent in females (70.4%, 50/71) than in males (31.8%, 7/22) (P = 0.001) (**Table 2**). The frequency of smokers 0 pack-years (68.8%, 53/77) among patients with tumours having EGFR mutations was significantly higher than that among smokers 0–30 pack-years (50.0%) and smokers ≥30 pack-years (0.0%) (P<0.001) (**Table 2**). EGFR mutations were more frequent in tumours with CTR ranging from 0.25 to 0.5 (85.7%, 12/14) and from 0.5 to 1 (66.7%, 20/30) than in those with CTR ranging from 0 to 0.25 (51.0%, 25/49) (P=0.048) (**Table 2**). There was no association between the frequency of EGFR mutations and lesion maximum diameter or pathological stage of lung cancers. Further analysis showed that CTR is significantly different between the 19del group and the EGFR wild type group (P=0.022), but except for the L858R (P=0.103).

**Table 2:** Frequency of EGFR, KRAS and TP53 mutations in relation to clinicopathological and imaging characteristics of 93 sMPLA.

	EGFR Mutation		P	KRAS Mutation		P	TP53 Mutation		P
Variable	Mutated	Wild		Mutated	Wild		Mutated	Wild	
Sex									
Female	50 (70.4%)	21 (29.6%)	0.001	4 (5.6%)	67 (94.4%)	0.003	15 (21.1%)	56 (78.9%)	1.000
Male	7 (31.8%)	15 (68.2%)		7 (31.8%)	15 (68.2%)		4 (18.2%)	18 (81.8%)	
Pack-years									
0	53 (68.8%)	24 (31.2%)	<0.001	5 (6.5%)	72 (93.5%)	0.002	15 (19.5%)	62 (80.5%)	0.678
0-30	4 (50.0%)	4 (50.0%)		2 (25.0%)	6 (75.0%)		2 (25.0%)	6 (75.0%)	
≥30	0 (0.0%)	8 (100.0%)		4 (50.0%)	4 (50.0%)		2 (25.0%)	6 (75.0%)	
Maximal tumour size, cm									
≤1	27 (51.9%)	25 (48.1%)	0.062	7 (13.5%)	45 (86.5%)	0.130	4 (7.7%)	48 (92.3%)	<0.001
1-2	19 (76.0%)	6 (24.0%)		1 (4.0%)	24 (96.0%)		5 (20.0%)	20 (80.0%)	
2-3	9 (81.8%)	2 (18.2%)		1 (9.1%)	10 (90.9%)		6 (54.5%)	5 (45.5%)	
>3	2 (40.0%)	3 (60.0%)		2 (40.0%)	3 (60.0%)		4 (80.0%)	1 (20.0%)	
Pathology									
MIA	14 (56.0%)	11 (44.0%)	0.381	4 (16.0%)	21 (84.0%)	0.649	1 (4.0%)	24 (96.0%)	<0.001
Lepidic	8 (88.9%)	1 (11.1%)		0 (0.0%)	9 (100.0%)		1 (11.1%)	8 (88.9%)	
Aci	14 (73.7%)	5 (26.3%)		2 (10.5%)	17 (89.5%)		9 (47.4%)	10 (52.6%)	
Pap	1 (100.0%)	0 (0.0%)		0 (0.0%)	1 (100.0%)		0 (0.0%)	1 (100.0%)	
MP	1 (50.0%)	1 (50.0%)		0 (0.0%)	2 (100.0%)		1 (50.0%)	1 (50.0%)	
Solid	2 (50.0%)	2 (50.0%)		1 (25.0%)	3 (75.0%)		4 (100.0%)	0 (0.0%)	
Mucinous	1 (100.0%)	0 (0.0%)		0 (0.0%)	1 (100.0%)		1 (100.0%)	0 (0.0%)	
CTR									
0-0.25	25 (51.0%)	24 (49.0%)	0.048	7 (14.3%)	42 (85.7%)	0.145	4 (8.2%)	45 (91.8%)	0.001
0.25-0.5	12 (85.7%)	2 (14.3%)		0 (0.0%)	14 (100.0%)		2 (14.3%)	12 (85.7%)	
0.5-1	20 (66.7%)	10 (33.3%)		4 (13.3%)	26 (86.7%)		13 (43.3%)	17 (56.7%)	
Pathological stage									
0	16 (50.0%)	16 (50.0%)	0.272	4 (12.5%)	28 (87.5%)	0.730	2 (6.3%)	30 (93.8%)	0.014
I	38 (67.9%)	18 (32.1%)		7 (12.5%)	49 (87.5%)		14 (25.0%)	42 (75.0%)	
II	2 (50.0%)	2 (50.0%)		0 (0.0%)	4 (100.0%)		2 (50.0%)	2 (50.0%)	
III	1 (100.0%)	0 (0.0%)		0 (0.0%)	1 (100.0%)		1 (100.0%)	0 (0.0%)	

KRAS mutations were more frequent in males (31.8%, 7/22) than in females (5.6%, 4/71) (P = 0.003) (**Table 2**). The frequency of smokers ≥30 pack-years (50.0%, 4/8) and smokers 0–30 pack-years (25.0%, 2/8) among patients with tumours having KRAS mutations was significantly higher than that among smokers 0 pack-years (6.5%, 5/77) (P=0.002) (**Table 2**). There was no association between the frequency of KRAS mutations and lesion maximum diameter, pathological stage, or CTR of tumours.

TP53 mutations were more frequent in III (100%, 1/1) and II (50.0%) than in I (25.0%) and 0 (6.3%) (P = 0.014) (**Table 2**). TP53 mutations were more frequent in tumours with CTR ranging from 0.5 to 1 (44.3%, 13/30) than in those with CTR ranging from 0.25 to 0.5 (14.3%, 2/14) and from 0 to 0.25 (8.2%, 4/49) (P=0.001) (**Table 2**). The frequency of TP53 mutations was 7.7% (4/52) in tumours sized ≤1 cm, 20.0% (5/25) in tumours sized >1 to ≤ 2 cm, 54.5% (6/11) in tumours sized >2 to ≤ 3 cm, and 80.0% (4/5) in tumours sized >3 cm. There was no association between the frequency of TP53 mutations and sex or smoker pack-years.

EGFR L858R mutations were higher in CTR ranging from 0.25 to 0.5 (77.8%, 7/9) than from 0.5 to 1 (47.4%, 9/19) and from 0 to 0.25 (38.5%, 15/39) (P = 0.103) (**Table 3**). The frequencies of EGER 19Del mutations and EGFR wild type were significantly different with lung adenocarcinoma with predominant ground -glass opacity. EGFR 19Del mutations were more frequent in tumours with CTR ranging from 0.25 to 0.5 (71.4%, 5/7) than in those with CTR ranging from 0.5 to 1 (41.2%, 7/17) and from 0 to 0.25 (20.0%, 6/30) (P=0.022) (**Table 3**).

**Table 3:** Association between the Proportion of EGFR mutation and CTR features.

Variable	L858R	Wild Type	P	19del	Wild Type	P
CTR						
0-0.25	15 (38.5%)	24 (61.5%)	0.103	6 (20.0%)	24 (80.0%)	0.022
0.25-0.5	7 (77.8%)	2 (22.2%)		5 (71.4%)	2 (28.6%)	
0.5-1	9 (47.4%)	10 (52.6%)		7 (41.2%)	10 (58.8%)	

### Mutation Spectra of Synchronous MPLAs

**Figure 2:**
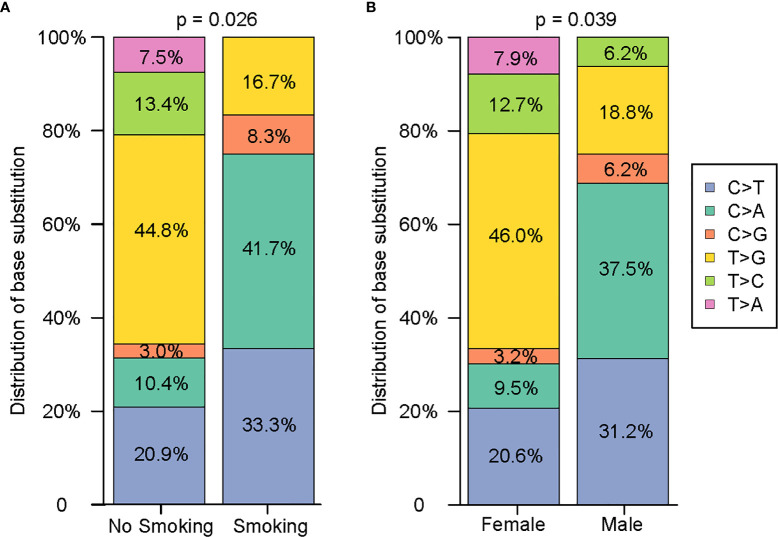
Comprehensive analysis of the distribution characteristics of 79 single nucleotides on the basis of smoking history and sex. **(A)** Comparison of the difference in the single nucleotide variants between nonsmokers and smokers. **(B)** Comparison of the difference in the single nucleotide variants between females and males.

Since NGS simultaneously assesses indels, rearrangements, single-nucleotide variants (SNVs), and copy number variations (CNVs), it facilitates the mechanistic study of intertumoural heterogeneity. Somatic mutations were identified by NGS in 93 samples from 42 patients with sMPLA (**Figure 1**). In this study, we identified 108 gene mutations, including 79 somatic single nucleotide variants (SNVs) and 29 indel mutations. Indel mutations were divided into 1 frameshift deletion; 1 frameshift substitution; 15 nonframeshift deletion; 4 nonframeshift insertion; 8 nonframeshift substitution. The 79 SNVs were divided into 1 stopgain and 78 nonsynonymous SNVs, the latter containing 12 C>A, 3 C>G, 18 C>T, 5 T>A, 9 T>C, 32 T>G. Mutational spectrum analysis revealed a strong enrichment of C > A transitions (41.7%, 5/12) and C > T transversions (33.3%, 4/12), which are associated with a history of smoking, whereas tumours from patients with no smoking history were more likely to have T > G transitions (44.8%, 30/67) (P = 0.026; **Figure 2**), indicating the impact of tobacco smoke on the mutational pattern during tumour progression. The correlation between the frequency of nonsynonymous SNVs and the sex classification of lung cancer patients is shown in **Figure 2**. Females were associated with a high frequency of T> G transversions (46.0%, 29/63), but C > A transitions (37.5%, 6/16) and C > T transversions (31.2%, 5/16) were more frequent in tumours from males (**Figure 2**) (P = 0.039).

### Marked Intertumoural Heterogeneity in sMPLA

**Figure 3:**
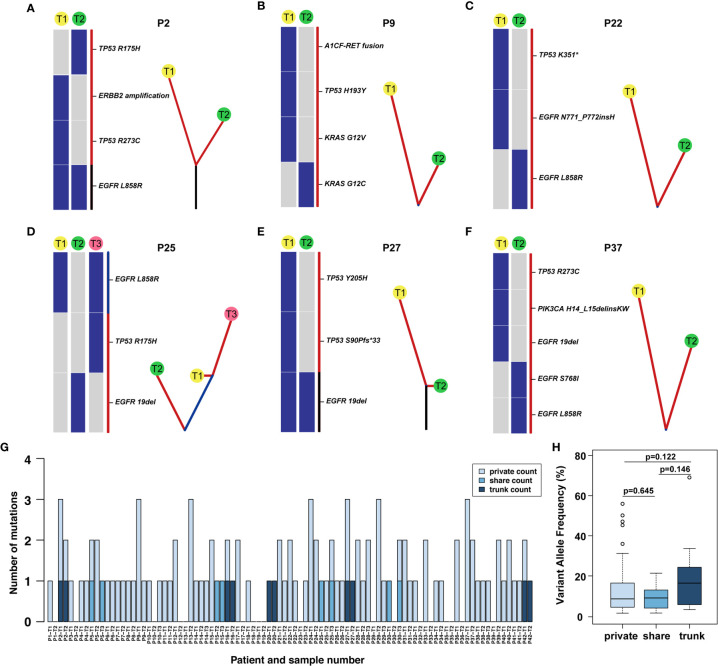
Phylogenetic tree and the distribution of trunk, shared, and private mutations. **(A–F)** The heat map on the left of each panel describes the regional distribution of all somatic mutations. The maximum parsimony algorithm was used to construct a phylogenetic tree for each patient on the right side of each panel. The mutation categories shown in the heat map are represented by the colour of each line, and the length of the trunk and branches are proportional to the number of mutations in each lesion. **(G)** The distribution of trunk, shared and branch mutations of 93 synchronous multiple primary lung adenocarcinoma lesions defined by next-generation sequencing. **(H)** The variant allele frequency (VAF) of the trunk, share and private mutations.

Among 42 patients with synchronous multiple primary lung adenocarcinoma, 7.1% (3/42) of patients had consistent gene status among tumours, and 92.9% (39/42) of patients had inconsistent gene status, suggesting that sMPLA has obvious genetic heterogeneity. The intertumoural gene status of 1 case was EGFR L858R mutation, and the remaining 2 cases were wild-type from P19 to P35. There were 2 tumours at P19, which were all adenocarcinomas *in situ* located on the same lobe. The two tumours of P35 were also all adenocarcinomas in situ, but the anatomical location was distinct. In case P9, T1 harboured KRAS p. G12 V, TP53 p. H193Y and A1CF-RET fusion mutations, whereas T2 had a KRAS p. G12C mutation, suggesting that these tumours may be driven by different molecular events (**Figure 3B**). In P25, T1 and T3 have a shared EGFR L858R mutation, and T3 also has a TP53 p. R175H, but T2 had an EGFR 19del mutation that was different from other lesions, indicating that the patient’s three lung adenocarcinomas were independent primary tumours (**Figure 3D**).

### The Distribution of Trunk, Shared Mutations, and Branch Mutations in the Phylogenetic Trees

Through multigene analysis of tumours, the phylogenetic tree can determine the evolution of each cancer genome, thereby revealing how multiple simultaneous primary lung adenocarcinomas have evolved. On the phylogenetic trees, each cluster corresponds to a tumour, and the private length of the corresponding tumour was related to the number of mutations. We further studied the intertumoural mutation spectra of each patient with sMPLA (**Figure 3**). Three types of somatic mutations were reflected in the phylogenetic tree model, including trunk mutations, which occur in all lesions, shared mutations, this mutation occurs in at least two lesions but not all lesions, and branch mutations, which are derived from a single lesion with private mutations. In **Supplementary Table 1**, we clearly show that 4 of the 42 sMPLA patients had both trunk and branch mutations, 4 patients had shared mutations, 1 patient had only trunk mutations, and the remaining patients had only private mutations. the number of trunk mutations, shared mutations, and branch mutations accounted for 4.95%, 3.96%, 91.09% of the phylogenetic trees, respectively. Further analysis showed that the variant allele frequency (VAF) of the trunk, share and private mutations. Notably, the VAFs of the trunk mutations (median value = 0.164) were higher than those of the shared (median value = 0.091) and private (median value = 0.086) mutations (**Figure 3H**). These data suggest that the trunk mutations occur earlier than the non-trunk mutations.

The somatic genetic alterations of synchronous lesions had the lowest proportion of trunk-shared mutations and the highest proportion of private mutations. In case P2, the T1 mucinous lesions had EGFR L858R, TP53 R273C mutation and ERBB2 amplification, yet the T2 solid lesions had EGFR L858R, TP53 R175H mutation (**Figure 3A**). Case P9-T1 had KRAS G12V, TP53 H193Y, A1CF-RET fusion in the solid predominant subtype, while the AIS was KRAS G12C in P9-T2 (**Figure 3B** and **Supplementary Table 1**). Case P25 had three lesions, both P25-T1 and P25-T3 had a shared mutation of EGFR L858R, and P25-T3 had a TP53 R175H mutation rather than P25-T1, and P25-T2 had EGFR 19del mutation (**Figure 3D**). Case 27 had an EGFR 19del trunk mutation, but P27-T1 also had TP53 Y205H, TP53 S90Pfs*33 mutation (**Figure 3E**).

Several driver mutations were subclonal and possibly occurred as late events in sMPLA, including mutations in BRAF, ERBB2, PIK3CA, and RET. In an *in vitro* study, PIK3CA mutation caused continuous activation of PI3K/Akt signal transduction, which caused EGFR 19del mutation HCC827 cells to develop resistance to gefitinib (16). Case P37-T1 had EGFR 19del, TP53 R273C, PIK3CA H14_L15delinsKW in acinar predominant subtype, while P37-T2 had EGFR L858R, EGFR S768I in MIA (**Figure 3F**). According to the distribution of gene mutations, 4 patients showed trunk mutations of EGFR L858R in P2, P16, P20, P42, and 1 patient showed trunk mutations of EGFR 19del in P27 (**Figure 3G** and **Supplementary Table 1**). Shared mutations were detected among different lesions from patients 5, 15, 25 and 30; Furthermore, the types of shared mutations in these patients were all EGFR L858R. In contrast, EGFR was a gene on the trunk of the phylogenetic tree that was mainly mutated in a completely cloned manner. This finding further shows that EGFR was an early mutation in the evolution of synchronous multiple primary lung adenocarcinoma (**Figure 3H**).

## Discussion

In this study, we comprehensively analysed the similarities in mutational status in a series of 93 tumours from 42 patients with surgically resected synchronous multiple primary lung adenocarcinomas, such as SNV, Indel mutations of EGFR, ALK, KRAS, BRAF, ERBB2, PIK3CA, and TP53, fusion mutations of ALK, ROS1, RET genes, MET jumping mutations and copy number variation of ERBB2, MET genes. In order to clarify the discrepancy of driver mutations between the synchronous lesions in sMPLA, it was important to define the lineage relationship of the synchronous tumours. By comparing the genomic features of synchronous lesions of sMPLA, we demonstrated a significant discordance rate of 92.9% (39/42) in a cohort of lung cancer patients with synchronous multiple primary lung adenocarcinomas.

Molecular classification relies on a common driver to determine whether synchronous lesions are from the same origin. The mutation prevalence observed in the sMPLA analysis appears to be different from other MPLC cohorts. The study found that EGFR mutations were observed in 61.3% (57/93) of tumours. However, EGFR mutations were higher than those of previous studies by Xiao et al. (17) and Zhu et al. (18). In the study by Xiao et al. (17), EGFR mutation was found in 29 of 64 tumours from 35 patients with synchronous MPLAs, most significantly the EGFR 19del and EGFR L858R mutation, identified sMPLA dependence on this pathway for growth and proliferation and appeared to sensitize tumours to the effects of a small molecule Epidermal growth factor receptor tyrosine kinase inhibitor (EGFR TKI). In general, the EGFR mutation frequency of synchronous MPLAs was higher than that of the single Asian lung adenocarcinoma population in the PIONEER study (51.4%) (19). In the study of Chen et al. (20), KRAS mutations were observed in 7.4% (2/27) of tumours analysed, which was lower than the 11.8% (11/93) detected in our studies. In our study, somatic mutations in TP53 were detected in 20.4% of tumours analysed, which was consistent with previous studies by Chen et al. (20). TP53 mutation was an interesting discriminating factor owing to its frequent involvement in lung cancer and to the absence of hotspot variants. TP53 mutation can transform cells into the cancer phenotype, desensitize targeted drugs, and increase genome heterogeneity, occurring at the onset or after subclonal diversification. Tumours with TP53 mutations have high heterogeneity, different pathological types and clinical stages, and unfavourable prognoses (21). In our study, BRAF mutations occurred in 6.5% of synchronous MPLAs, yet the most common mutation, BRAF V600E, was observed in 1.1% of synchronous MPLAs. ALK, ROS1, MET mutations were not identified. KRAS and EGFR mutations are usually mutually exclusive, but when they coexist, KRAS mutations may lead to resistance to EGFR inhibitors (22).

In our study, we analysed the clinicopathological and imaging characteristics of the lesions to determine the factors that may predict EGFR and TP53 inactivation mutations. There are significant differences in CTR, gender and smoking history between EGFR mutant and EGFR wild-type adenocarcinoma. The frequency of EGFR mutations in the CTR ranging from 0.25 to 0.5 group was significantly higher than that in the CTR ranging from 0 to 0.25 group, and with the increase of solid components, there was a downward trend in the CTR from 0.5 to 1 group. Further analysis showed that the prevalence of L858R was higher than 19del, and most L858R mutation lesions presented as ground-glass opacity (GGO). Lee et al. (23) surveyed GGO volume percentage in tumors with L858R mutation was significantly higher than that in EGFR wild-type tumors (P < 0.001) and 19del mutated tumors (P < 0.001). Contrary to previous research (24), we found that GGO proportion is significantly different between the 19del group and the EGFR wild type group, but except for the L858R. In our study, TP53 mutation was detected more frequently in higher CTR lesions than in lower CTR lesions. The maximal diameter of tumour in the TP53 mutation group was significantly larger than that in the wild-type group. Moreover, among the lesions with maximal diameter >2 cm and CTR ranging from 0.5 to 1, TP53 mutations were found more frequently than in the other groups, and it is often accompanied by EGFR mutation. TP53 inactivating mutations may be involved in the process of tumour progression and may promote the transformation of tumours to greater malignant potential. TP53 mutation was involved in the consolidation of the central area of adenocarcinoma, EGFR may be associated with the appearance of central consolidation.

Understanding the pathogenesis and evolutionary biological basis of these synchronous lesions in sMPLA might guide therapy and improve prognosis. Genotype-matched precision medicine needs to understand the biological basis of intratumour or intertumoural heterogeneity and genomic instability during cancer evolution and the mutational processes within the tumours and their dynamics change over time (25). Intertumoural heterogeneity was described as molecular genetic differences between tumours from different sites in the same patient or from completely different patients. The heterogeneity of tumour evolution, both over time within a tumour and spatially between different primary and metastatic sites, raises the question of optimally defining the molecular status of a tumour and best incorporating the understanding of this heterogeneity into treatment strategies (26). According to the mutation copy number and the cancer cell fraction, mutations were classified into “early events” or “late events”. McGranahan et al. concluded that clonal mutations represent mainly early events in the process of tumour evolution, while late mutation events were subclonal mutations or occurred after genome doubling or amplification events (27). Late mutation events mainly occurring on the branches of a tumour’s phylogenetic tree led to cancer heterogeneity, plasticity and drug resistance, which usually leads to clinical complications and adverse side effects (28). Later somatic events are heterogeneous and exist in the subclones that drive tumour progression (29). In our study, approximately 91.09% of driver mutations were branched or subcloned, including those in genes such as PIK3CA, KRAS, and TP53, indicating that these driver mutations were relatively late events. This study is consistent with the results of previous studies (30). EGFR mutations were mainly trunked, which means that EGFR driver mutations may be defined as early events involved in the early tumorigenesis of multiple primary lung cancers before clonal expansion. Previous studies have suggested that TP53 may be one of the founder mutations (25), but TP53 mutations were mainly branched and may play a role in the maintenance and progression of sMPLA in our study. Zhao et al. (31) demonstrated that TP53 mutation affects the treatment response of targeted therapy EGFR TKIs when coexisting with EGFR 19del or EGFR L858R. Therefore, it was understandable that rapid and significant tumour regression and other clinical benefits can be obtained by targeting these alterations, but other genetic changes were more common in a single branch of the tumour evolutionary tree.

In conclusion, this study performed next-generation sequencing on postoperative lesions of sMPLA and confirmed that they had high genetic heterogeneity, and most of the mutations were branched mutations, indicating that most of the lesions in patients with sMPLA have different cell origins. Although we do not know how sensitive the targeted therapy is in the treatment of sMPLA, the high genetic heterogeneity observed in this study predicts that the overall therapeutic effect of targeted therapy for synchronous multiple primary lung adenocarcinomas is poor. Targeting driver mutations in some of the tumour cells of the lesion may only affect the growth of tumours in that area but may not have any effect on other types of mutations, resulting in limited clinical benefits. To make matters worse, targeted therapy may have contradictory stimulatory effects on subclones lacking corresponding mutations and further promote tumour growth. Therefore, we need to accurately understand the genetic status of each lesion and evaluate intertumoural genetic heterogeneity to provide guidance for subsequent treatment. We found that clinicopathological and imaging characteristics of lesions in patients with sMPLA could assist to predict the mutation status of EGFR, TP53 and KRAS, and provide direction for precise treatment strategies for synchronous multiple primary lung adenocarcinoma. There are some limitations in the present study that are worthy of our consideration. The sample size of our study is relatively small, and retrospective studies may lead to selection bias. Further prospective cohort studies on plenty of patients are warranted.

## Data Availability Statement

The datasets presented in this study can be found in online repositories. The names of the repository/repositories and accession number(s) can be found below: https://ngdc.cncb.ac.cn/gsa-human/browse/HRA001237.

## Ethics Statement

The studies involving human participants were reviewed and approved by Ethics Committee of Tumor Hospital affiliated to Shandong First Medical University. The patients/participants provided their written informed consent to participate in this study.

## Author Contributions

QZ and HJ contributed equally conceived research ideas, designed analysis methodology, and collected and sorted out raw data. LH and ZW wrote the initial draft. SH, HH, and AY prepared the figure and all authors contributed to the analysis of the results. PS supervised the research and provided financial support for the project. All authors contributed to the article and approved the submitted version.

## Funding

The name of granting agencies: Development Center for Medical Science & Technology National Health Commission of the People’s Republic of China Grant numbers: WA2021RW02 Funder’s role: (1) Carry out research on major policies, strategies and plans for the development of health science and technology to provide support for the government’s macro decision-making; (2) Undertake the project management and other related functions of the national science and technology plan entrusted by the National Health Commission; (3) Assist in the improvement of health and health The field scientific research management support system provides technical and management support for the government to implement related management; (4) Carrying out related exchanges, seminars, training and science popularization of scientific and technological progress in the health and health field; (5) Promoting the transfer and transformation of health and health science and technology achievements and industries (6) Carrying out international exchanges and cooperation in the field of health science and technology; (7) Undertaking other tasks assigned by the National Health Commission. The authors declare that this study received funding from Development Center for Medical Science & Technology National Health Commission of the People’s Republic of China. The funder had the following involvement in the study: Intertumoural Heterogeneity and Branch Evolution of Synchronous Multiple Primary Lung Adenocarcinomas by Next-Generation Sequencing Analysis.

## Conflict of Interest

Authors HH and AY were employed by the company Berry Oncology Corporation.

The remaining authors declare that the research was conducted in the absence of any commercial or financial relationships that could be construed as a potential conflict of interest.

## Publisher’s Note

All claims expressed in this article are solely those of the authors and do not necessarily represent those of their affiliated organizations, or those of the publisher, the editors and the reviewers. Any product that may be evaluated in this article, or claim that may be made by its manufacturer, is not guaranteed or endorsed by the publisher.

## Supplementary Material

Supplementary Table 1 Detailed mutational landscape, clinicopathological and imaging information of 75 mutated sMPLA tumours.

Supplementary Table 2 Detailed molecular landscape for each sample of 42 sMPLA cases.
